# The Richmond Hospital

**Published:** 1893-05-06

**Authors:** 


### THE RICHMOND HOSPITAL.

A dinner in aid of the "Princess May's Ward for Children" was held at the Star and Garter Hotel, Richmond, on the 27th ult. The Duke of Cambridge presided. Amongst those present were the Duke of Teck, Sir J. Whittaker Ellis, Sir F. Dixon-Hartland, M.P., Mr. Justice Hawkins, Sir E. Saunders, Sir Horace Farquhar, the Mayor of Richmond, and the Vicar of Richmond. Ladies were present in the gallery, and amongst them were the Duchess of Teck and the Princess May. The Duke of Cambridge mentioned that the Queen had consented to give her patronage to the hospital at the request of Princess May. He said that he had presided at dinners in aid of the hospital on two former occasions. In 1875, the first occasion on which he presided, they desired to increase the very small hospital then existing, which contained only 12 beds. In 1882 the number of beds had increased to 40. A children's ward it was felt would be a very great benefit. It would need £5,000 to achieve this object. Mr. Charrington had subscribed £1,000 to endow one cot, and it was hoped his example would be followed. During the evening subscriptions and donations to the amount of £2,838 were announced, this sum including £1,000 subscribed by Mr. Charrington, and £671 collected by the Duchess of Teck and the Princess May.

